# Westminster Hospital's Special Appeal

**Published:** 1917-12-01

**Authors:** J. Wolfe Barry

**Affiliations:** Chairman House Committee and Chairman Special Appeal. Westminster Hospital, Broad Sanctuary, S. W. 1


### THE EDITOR'S LETTER-BOX.

### Westminster Hospital's Special Appeal.

*To the Editor of* The Hospital.

Sir,—Even in these days of great events and small newspapers I trust you will find room for a very brief statement of why Westminster Hospital finds it necessary to ask for immediate financial assistance. A full statement will gladly be sent to anyone applying for it.

Westminster Hospital began its work for the sick poor so long ago as 1719. Its record for almost two centuries is a good one, and it has become an integral and important part of that splendid hospital system of which London is proud. Summarised, the present financial position of the hospital is as follows :—

1. Its bank overdraft is £5,500.

2. To pay off overdue tradesmen's accounts the small capital fund of the hospital has recently been appreciably reduced by the sale of securities.

3. Despite all possible care and economy, the annual expenditure has grown from £21,000 in 1913 to £29,000 in 1917, while the income has been seriously diminished. The War Office payment in respect of 100 beds for wounded soldiers treated in the hospital falls far short of the rise in expenditure due to the rise of prices during the war.

4. The annual subscription list yields so small an amount that unless it be increased by several thousands there must necessarily be recurrent financial crises similar to that which exists at present.

To place the finances of the hospital on a satisfactory and secure basis (1) a sum of at least £20,000 is wanted to clear off its present and immediate liabilities, and (2) such an increase in the sum derived from annual subscriptions as will enable future yearly expenditure to be met out of future yearly income. His Royal Highness the Prince of Wales, the president of the hospital, has been pleased to contribute the sum of £1,000, and a preliminary list of other donors will shortly be issued. Donations and subscriptions in response to this special appeal will be gladly received by the treasurers or myself at the hospital. In particular I ask for help from the very many to whom the name of Westminster has special associations of any sort or kind.—Yours, etc., J. Wolfe Barry,

Chairman House Committee
and Chairman Special Appeal.

Westminster Hospital, Broad Sanctuary, S. W. 1,
November 27, 1917.

### "Is Thy Secretary a Dog?"

*To the Editor of* The Hospital.

